# Paraquat dichloride: MAK Value Documentation, addendum – Translation of the German version from 2023

**DOI:** 10.34865/mb191042e10_2ad

**Published:** 2025-06-30

**Authors:** Andrea Hartwig, MAK Commission

**Affiliations:** 1 Institute of Applied Biosciences. Department of Food Chemistry and Toxicology. Karlsruhe Institute of Technology (KIT) Adenauerring 20a, Building 50.41 76131 Karlsruhe Germany; 2 Permanent Senate Commission for the Investigation of Health Hazards of Chemical Compounds in the Work Area. Deutsche Forschungsgemeinschaft, Kennedyallee 40, 53175 Bonn, Germany. Further information: Permanent Senate Commission for the Investigation of Health Hazards of Chemical Compounds in the Work Area | DFG

**Keywords:** Paraquatdichlorid, Pestizid, Herbizid, Toxizität, Bewertung, paraquat dichloride, pesticide, herbicide, toxicity, evaluation

## Abstract

Paraquat dichloride [1910-42-5] is used as a herbicide but is no longer approved in the European Union. The previous MAK value documentations and addenda do not reflect the current data situation of the substance. The MAK Commission decided that a new evaluation is not of high priority. The MAK value and the other classifications are therefore suspended and the substance is listed in the Section II c of the List of MAK and BAT Values for substances no longer evaluated.

**Table:** 

**MAK value**	**see Section II c of the List of MAK and BAT Values**
**Peak limitation**	**–**
	
**Absorption through the skin**	**–**
**Sensitization**	**–**
**Carcinogenicity**	**–**
**Prenatal toxicity**	**–**
**Germ cell mutagenicity**	**–**
	
**BAT value**	**–**
	
Synonyms	1,1′-dimethyl-4,4′-bipyridiniumdichloride methyl viologen paraquat
Chemical name (IUPAC)	1-methyl-4-(1-methylpyridin-1-ium-4-yl)pyridin-1-ium;dichloride
CAS number	paraquat ion: 4685-14-7 paraquat dichloride: 1910-42-5
Molecular formula	C_12_H_14_Cl_2_N_2_
Molar mass	257.16 g/mol
Melting point	decomposes on heating (IFA 2022)
Vapour pressure at 25 °C	0 Pa (ECHA 2022)
log K_OW_ at 20 °C	–4.5 (ECHA 2022)
Solubility	620 g/l water (IFA 2022)

This addendum was prepared because the published evaluation no longer reflects the data currently available for the MAK value and for the designation and classification of the substance.

The name paraquat refers to both the cation itself and the dichloride salt.

Paraquat dichloride is a herbicide used in many areas of agriculture to control broad-leaved plants. It readily undergoes reduction followed by oxidation, thereby generating free radicals. In 1973, a MAK value of 0.1 mg/m^3^ I was established for the substance. In the addendum published in 2000, paraquat dichloride was classified in Peak Limitation Category I with an excursion factor of 1. In 1983, it was designated with an “H” (for substances which can be absorbed through the skin in toxicologically relevant amounts) (Greim 2000, available in German only; Henschler 1973, available in German only).

In the European Union, paraquat salts are not approved for use as herbicides according to Regulation (EC) No 1107/2009 concerning the placing of plant protection products on the market (European Commission 2023; European Parliament and European Council 2009). The substance is still in use in many other countries such as the United States, Japan, Australia and China (AERU 2022 a, b). In the Federal Republic of Germany, paraquat dichloride was approved for use as herbicide in 1971. Approval has been suspended since July 2007. The last authorization was revoked in December 2008. In the former German Democratic Republic, approval was granted even before 1966 (BVL 2010). Paraquat dichloride is on the list of chemicals in Annex I Parts 1 and 2 of the PIC (Prior Informed Consent) Regulation (EU) No 649/2012 (European Commission 2022). Exporters are thus required to submit notification of their intention to export this substance and receive explicit consent from the importing country prior to export.

The previous evaluation does not reflect the currently available data. However, a re-evaluation of the substance is not a priority. Therefore, the MAK value, the peak limitation and the “H” designation have been withdrawn and the substance has been allocated to Section II c of the List of MAK and BAT Values (DFG 2022). This section lists substances for which the previous MAK values, designations and classifications have been withdrawn and which are no longer being reviewed at present.

## References

[ref_N68SPHW9] (2022). Paraquat (Ref: PP 148). Pesticides Properties DataBase.

[ref_R59JVE7N] (2022). Paraquat dichloride. Pesticides Properties DataBase.

[ref_B3P3UERE] Bundesamt für Verbraucherschutz und LebensmittelsicherheitBVL (2010). Berichte zu Pflanzenschutzmitteln 2009. Wirkstoffe in Pflanzenschutzmitteln – Zulassungshistorie und Regelungen der Pflanzenschutz-Anwendungsverordnung.

[ref_ADQSLACU] Deutsche ForschungsgemeinschaftDFG (2022). List of MAK and BAT Values 2022. Maximum Concentrations and Biological Tolerance Values at the Workplace. Permanent Senate Commission for the Investigation of Health Hazards of Chemical Compounds in the Work Area, report 58.

[ref_E5ZGDWBL] (2022). Paraquat-dichloride. Brief profile, last updated 17 Nov 2022.

[ref_3ZNLNEA6] European CommissionEC (2022). Commission Delegated Regulation (EU) 2022/643 of 10 February 2022 amending Regulation (EU) No 649/2012 of the European Parliament and of the Council as regards the listing of pesticides, industrial chemicals, persistent organic pollutants and mercury and an update of customs codes. OJ L.

[ref_A4SWKWCT] European CommissionEC (2023). Paraquat. EU Pesticides Database (v.2.2) active substances.

[ref_KX39KWS7] European Parliament, European Council (2009). Regulation (EC) No 1107/2009 of the European Parliament and of the Council of 21 October 2009 concerning the placing of plant protection products on the market and repealing Council Directives 79/117/EEC and 91/414/EEC. OJ L.

[ref_49BXHB4Q] Greim H (2000).

[ref_EQNL7RMC] Henschler D (1973).

[ref_3NAV6EUK] (2022). 1,1′-Dimethyl-4,4′-bipyridynium dichloride. GESTIS-Stoffdatenbank.

